# Spatial distribution of entomopathogenic nematodes and their relationship with soil chemical attributes in tropical agroecosystems

**DOI:** 10.1007/s10661-026-15875-0

**Published:** 2026-09-04

**Authors:** Nádia Souza, Vanessa Andaló, Luziane Ribeiro Indjai, George Deroco Martins, Lucas Silva de Faria, Gleice Aparecida de Assis

**Affiliations:** Federal University of Uberlândia, Monte Carmelo Campus, Monte Carmelo, MG 38500-000 Brazil

**Keywords:** Biological control, Soil microbiome, Rhizosphere interactions, Geospatial analysis, Micronutrients, Ecosystem functioning

## Abstract

The distribution of entomopathogenic nematodes (EPNs) in agricultural soils is influenced by complex interactions between edaphic factors and crop management practices, yet these relationships remain poorly understood under tropical conditions. This study evaluated the spatial distribution of EPNs and their association with soil chemical attributes in five crop systems in southeastern Brazil. A total of 82 soil samples were collected and analyzed using insect-baiting techniques and geospatial tools, including kernel density and interpolation methods. EPN occurrence varied across crops and was positively associated with higher concentrations of potassium and iron, while an inverse pattern was observed for copper. These results suggest that micronutrients may indirectly influence EPN distribution by modulating soil microbial communities and plant–soil interactions. Although causal relationships cannot be definitively established, the spatial patterns identified highlight the importance of soil chemical composition in structuring beneficial soil fauna. The integration of geospatial analysis provides a valuable framework for optimizing the application of biological control agents in Integrated Pest Management (IPM) programs. These findings contribute to a better understanding of soil ecological dynamics and support the development of more sustainable agricultural practices.

## Introduction

The expansion of agricultural production has led to the intensive use of pesticides to control pests, diseases, and weeds. Although effective, the excessive reliance on chemical inputs has raised serious environmental and health concerns, including soil contamination, biodiversity loss, and disruption of beneficial microbial communities. In agricultural soils, such impacts can alter microbial structure and ecosystem functioning, ultimately affecting soil health and sustainability (Campillo-Cora et al., 2025; Navarrete et al., 2017).

Entomopathogenic nematodes (EPNs) have emerged as a promising alternative for sustainable pest management. Species from the genera *Steinernema* and *Heterorhabditis*, in association with symbiotic bacteria (*Xenorhabdus* and *Photorhabdus*), act as efficient biological control agents against a wide range of soil-dwelling insect pests. Their infective juvenile (IJ) stage can locate and kill hosts within a short period, making them suitable for integration into Integrated Pest Management (IPM) programs (Abd-Elgawad, 2023; Lu et al., 2016).

Despite their potential, the success of EPNs under field conditions remains inconsistent and is strongly influenced by environmental factors, particularly soil properties. Previous studies have demonstrated that variables such as temperature, moisture, and soil texture affect EPN persistence and performance, but the role of soil chemical attributes in shaping their spatial distribution is still poorly understood, especially under tropical agricultural systems (Campos-Herrera & Turlings, 2015).

In addition, increasing evidence suggests that soil nutrients may indirectly influence EPN populations by modulating microbial communities and plant–soil interactions. Micronutrients such as iron and copper play key roles in microbial competition and metabolism, including siderophore production, which can affect microbial community structure and ecological interactions in the soil (Gu et al., 2020; Hu et al., 2024). These processes may have cascading effects on soil fauna, including entomopathogenic nematodes, yet remain largely unexplored in field-based spatial studies.

Despite the recognized importance of entomopathogenic nematodes as biological control agents, their distribution within agricultural fields is rarely homogeneous. Understanding their spatial distribution is essential for identifying zones with greater natural biological control potential, optimizing sampling strategies, supporting site-specific management practices, and improving the efficiency of Integrated Pest Management (IPM) programs. Geospatial approaches also provide a framework for linking biological patterns with environmental variability, allowing a better understanding of the ecological factors that regulate EPN occurrence under field conditions.

Therefore, this study aimed to evaluate the spatial distribution of entomopathogenic nematodes and investigate potential associations with soil chemical attributes in different crop systems. By integrating field sampling with geospatial analysis, this study contributes to a better understanding of soil ecological interactions and to support the development of more efficient biological control strategies in tropical agroecosystems.

## Materials and methods

### Study area description

Soil samples for EPN isolation were collected from agricultural areas on the Pastão and Londrina farms, both located in the municipality of Monte Carmelo, Minas Gerais, in the microregion of the Triângulo Mineiro and Alto Paranaíba.

**Fig. 1:**
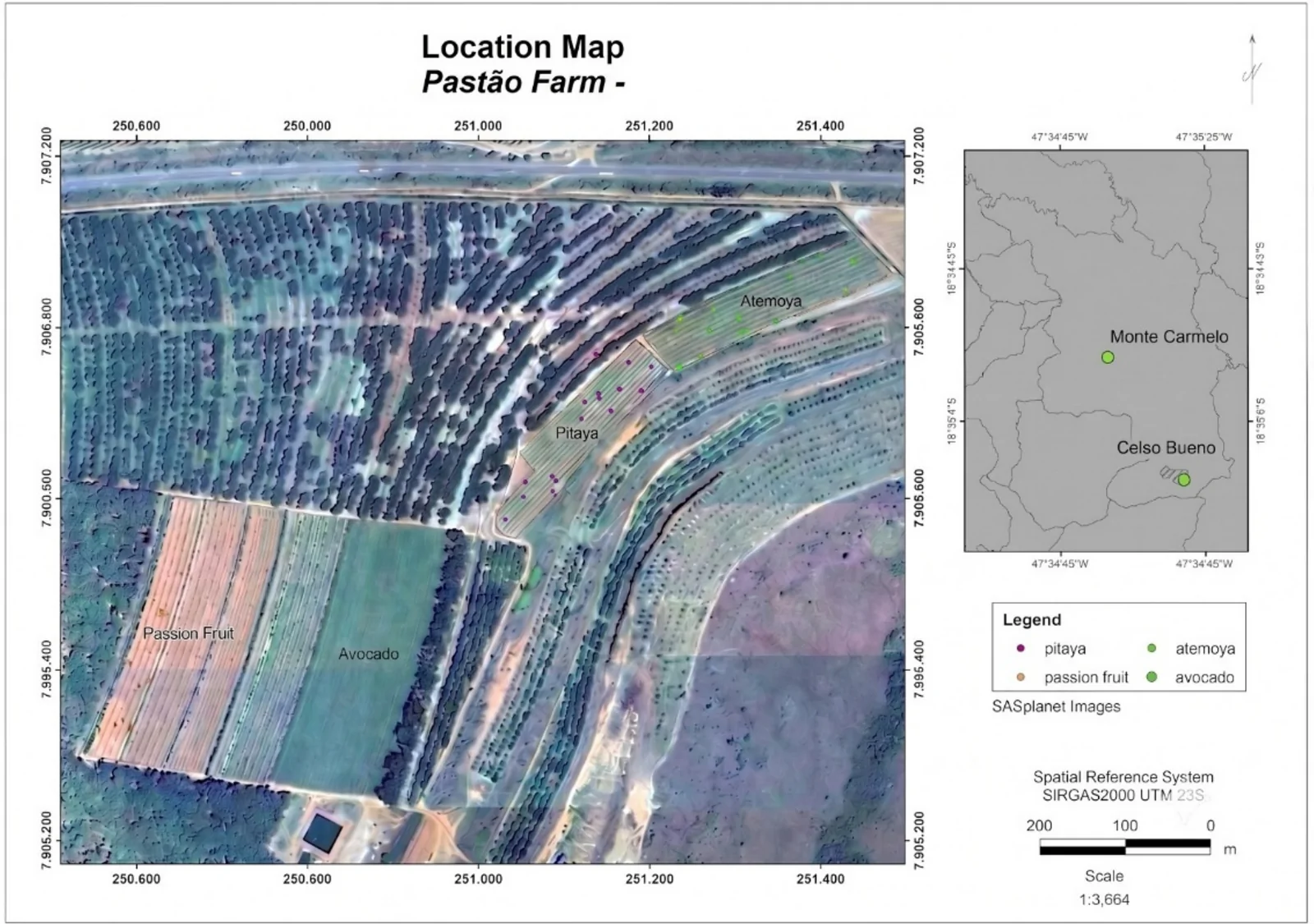
Location map of Pastão farm

**Fig. 2:**
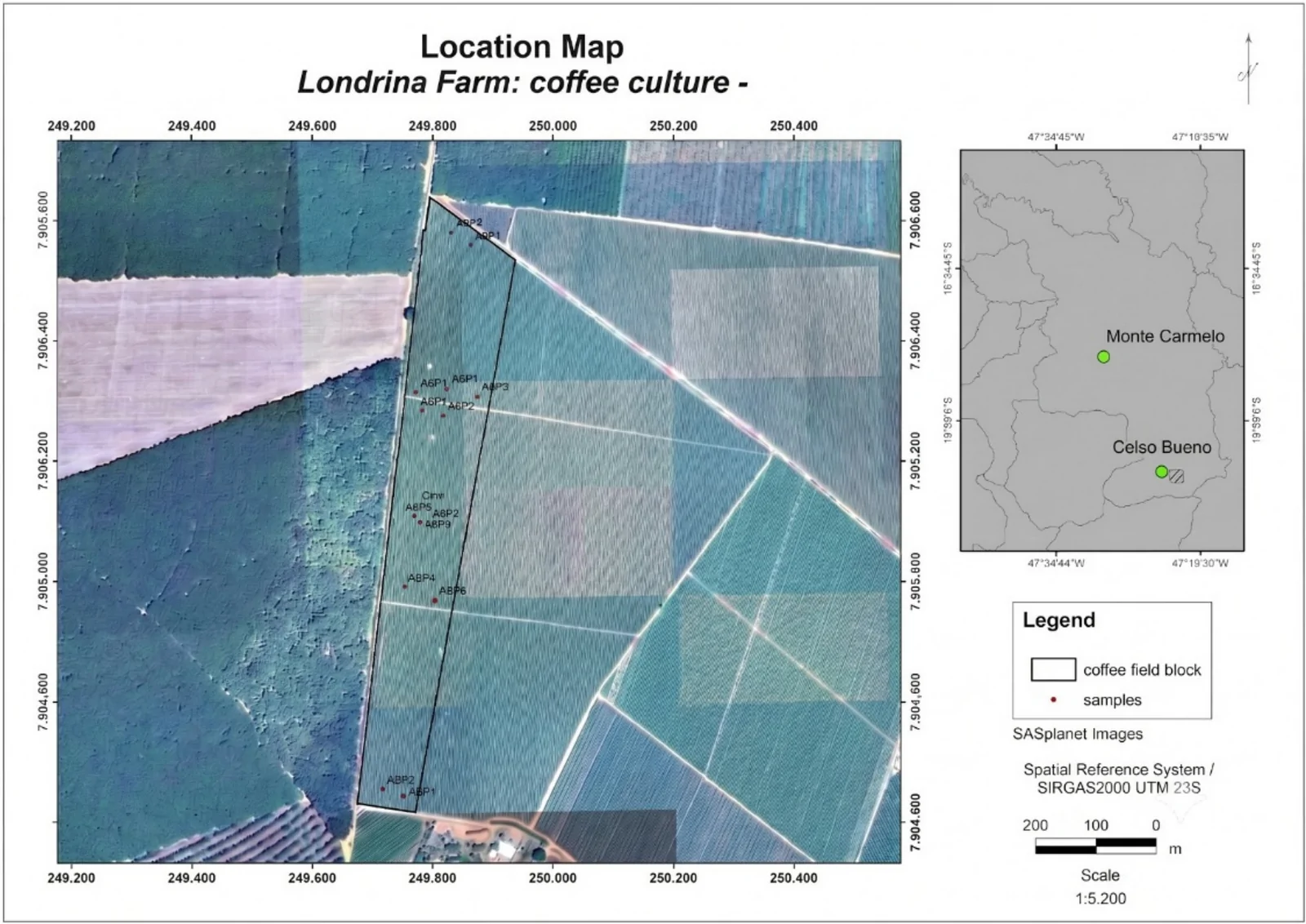
Location map of Londrina farm

The Pastão farm is situated at an altitude of 1015 m, at latitude 18° 55′ 20″ S and longitude 47° 21′ 55″ W, while the Londrina farm is located at an altitude of 1045 m, at latitude 18° 56′ 15″ S and longitude 47° 22′ 34″ W. The distance between the sampling locations on the two farms is approximately 2 km (Figs. 1 and 2).

The soil in the experimental areas is classified as Dystrophic Red Latosol, according to the Brazilian Soil Classification System (SiBCS). The climate is classified as Aw, according to Köppen. The region has a tropical climate with higher rainfall during the summer season.

Samples were collected between January and May 2024 in cultivated areas of avocado (*Persea americana*), atemoya (Annona × atemoya), passion fruit (*Passiflora edulis*), and pitaya (*Hylocereus undatus*) on the Pastão farm, and coffee (*Coffea arabica*) on the Londrina farm, totaling five cropping systems and 82 samples. Seventeen samples were obtained from each of the atemoya, pitaya, avocado, and passion fruit fields, corresponding to cultivated areas of approximately 2 ha, 1.5 ha, 2 ha, and 2 ha, respectively. In the coffee crop, 14 samples were collected, corresponding to cultivated areas of 13 ha. Although sampling density varied among cropping systems due to differences in field size, the study focused on exploratory spatial visualization and statistical associations rather than on detailed geostatistical modeling. Therefore, the sampling scheme was considered adequate to characterize broad patterns of EPN occurrence and its relationship with soil chemical attributes. Nevertheless, the lower sampling density in the coffee area may have reduced the detection of fine-scale spatial heterogeneity.

Cultural practices followed the conventional protocols adopted by each farm for each respective crop. Crop management practices at the Pastão farm varied throughout the sampling period. In the atemoya crop, soil microorganisms were applied in January, followed by mineral fertilization in February and March. In April, a biological insecticide and a biological nematicide were applied, and in May, a microbiological fungicide was used. In pitaya, soil microorganisms were applied in January and mineral fertilization in February, with no additional practices reported from March to May. For avocado, mineral fertilization was performed in January, with no treatments recorded in February and March. Soil microorganisms were applied in April, and no practices were reported in May (Table 1).

**Table 1:** Crop management practices adopted in atemoya, avocado, pitaya, and passion fruit orchards at Pastão Farm

Crop	January	February	March	April	May
Atemoya	Soil microorganisms	Mineral fertilization	Mineral fertilization	Biological insecticide; Biological nematicide	Microbiological fungicide
Pitaya	Soil microorganisms	Mineral fertilization	–	–	–
Avocado	Mineral fertilization	–	–	Soil microorganisms	–
Passion fruit	Soil microorganisms	Mineral fertilization	Fertigation	–	Mineral fertilization; Fertigation

January: *Atemoya*—soil microorganisms/*Pitaya*—soil microorganisms/*Avocado*—mineral fertilization (K)/*Passion fruit*—soil microorganisms. February: *Atemoya*—mineral fertilization (K)/*Pitaya*—mineral fertilization (NPK, NKS); K fertigation/*Passion fruit*—mineral fertilization (K, NPK, NKS, MAP). March: *Atemoya*—mineral fertilization (K)/*Passion fruit*—fertigation (NPK, K, N, Ca). April: *Atemoya*—biological insecticide/acaricide (*Beauveria bassiana*); microbiological nematicide (*Bacillus subtilis*, *Bacillus licheniformis*, and *Paecilomyces lilacinus*)/*Avocado*—soil microorganisms. May: *Atemoya*—microbiological fungicide (*Bacillus amyloliquefaciens* and *Trichoderma harzianum*)/*Passion fruit*—mineral fertilization (NPK); fertigation; growth regulator (ethephon)

In the passion fruit crop, soil microorganisms were applied in January, mineral fertilization in February, and fertigation in March. No practices were recorded in April, while both mineral fertilization and fertigation were carried out in May.

At the Londrina farm, management practices in the coffee crop included insecticide application in January, foliar fertilization in February, and fungicide application in March. No management practices were recorded in April. In May, combined applications of fungicide, insecticide/acaricide, foliar fertilization, and mineral fertilization were performed (Table 2).

**Table 2:** Crop management practices in the coffee plantation at Londrina Farm

Crop	January	February	March	April	May
Coffee	Insecticide	Foliar fertilizer	Fungicide	–	Fungicide; Insecticide/Acaricide; Foliar fertilization; Mineral fertilization

January: *Coffee*—insecticide (ketoenol); fungicide (strobilurin and triazole); foliar fertilizer (Zn and B); adjuvant. February: *Coffee*—foliar fertilization. March: *Coffee*—fungicide (strobilurin and triazole); insecticide (thiamethoxam and chlorantraniliprole); insecticide/acaricide/nematicide (avermectin); adjuvant; foliar fertilization (Zn and B); mineral fertilization (N). April: no management practices were recorded. May: *Coffee*—fungicide application; insecticide/acaricide application; foliar fertilization; mineral fertilization

The study was designed as an exploratory observational survey using spatially distributed sampling points across five crop systems. Each sampling point was considered an independent experimental unit. Sampling locations were randomly selected within each crop area while avoiding previously sampled sites to minimize spatial dependence.

### Soil sampling and processing

Approximately 400 g of soil was collected per sampling point, composed of three subsamples collected within a radius of 20 to 40 m. Geographic coordinates of each sampling point were recorded using a handheld GPS unit (Garmin GPSMAP 78, Garmin Ltd., USA), with an estimated positional accuracy of ±3–5 m under field conditions, referenced to the WGS84 coordinate system.

At each sampling point, the surface layer (0–5 cm) of soil was discarded, and the 5–15 cm layer was collected. During each sampling period, three to four random points were chosen per crop, avoiding previously sampled sites. Samples were collected in the morning every 15 to 21 days, prioritizing cooler hours to maintain sample integrity during transport. To reduce potential sampling bias, collections were conducted under similar climatic conditions and at regular intervals. Previously sampled points were excluded from subsequent sampling events to minimize spatial autocorrelation. The sampling schedule and number of samples per session were designed to optimize laboratory processing.

Soil samples were stored in 1-L polyethylene bags, labeled, placed in coolers, and transported to the Agricultural Entomology Laboratory (LABEN) at the Federal University of Uberlândia, Monte Carmelo campus. In the laboratory, entomopathogenic nematodes were isolated using the insect-baiting technique. Samples were placed into 500-mL polyethylene containers, each with six *Tenebrio molitor* L. (Coleoptera: Tenebrionidae) mid-instar larvae and maintained at room temperature under laboratory conditions. Containers were sealed and inverted (lid facing down). After 5 to 7 days, dead larvae showing symptoms of EPN infection were surface sterilized with 0.1% sodium hypochlorite and transferred to dry chambers (9-cm Petri dishes with filter paper). After 3 days, cadavers were moved to White traps (White, 1927) for the collection of IJs. Within approximately 8 days, IJs migrated from the cadavers to the water reservoir via the filter paper.

The pathogenicity of collected IJs was confirmed using Koch’s postulates by re-infecting healthy larvae and re-isolating the nematodes from newly infected hosts. The IJs were then preserved in a B.O.D. chamber at 16 ± 2 °C for 2 to 3 months. Following storage, they were re-inoculated into *T. molitor* larvae for multiplication and stored again under controlled temperature conditions.

### Map generation and spatial analysis

Chemical soil attributes analyzed included calcium (Ca), magnesium (Mg), potassium (K), manganese (Mn), sulfur (S), boron (B), iron (Fe), copper (Cu), and zinc (Zn). Soil analyses for the Pastão farm were conducted by Grupo Safrar, and those for the Londrina farm by the Unithal laboratory. Although chemical attributes were the primary focus, additional soil properties such as pH, organic matter content, and texture are recognized as important drivers of nematode distribution and should be considered when interpreting the results.

The objective of mapping was to visualize the spatial distribution of EPNs across the cultivation plots and explore potential correlations. All maps were generated using ArcGIS Desktop 10.0 with the Spatial Analyst extension, at the SIGEO Laboratory of Geodesy and Cartography Engineering at UFU.

A binary “presence/absence” variable (1 = present, 0 = absent) was assigned to each sample point. A heatmap was then generated using kernel density estimation to create a smoothed surface representing the influence of EPN presence in the surrounding area. Kernel density estimation was performed using a search radius of 5 m and a cell size of 5 m. These parameters were selected according to the spatial distribution and density of the sampling points, aiming to preserve local spatial variability while avoiding excessive smoothing of the resulting surface. Considering the relatively small size of the experimental fields and the exploratory nature of the spatial analysis, this configuration provided an appropriate balance between spatial detail and surface continuity for identifying areas with greater EPN occurrence. The resulting maps were divided into five classes ranging from 0 to 100, indicating the relative density of EPNs. Due to the extent of the study area, two separate maps were created: one for atemoya, pitaya, avocado, and passion fruit, and another for coffee. Kernel density estimation was used to identify hotspots of EPN occurrence, providing a smoothed spatial representation of presence points and allowing the identification of areas with relatively higher EPN occurrence across the study sites.

For soil attribute mapping, individual surfaces were created for each variable using the Topo to Raster tool. The Topo to Raster method was selected due to its ability to generate hydrologically consistent continuous surfaces from irregularly distributed environmental data, which is common in field-based soil studies. From these, contour lines were extracted and overlaid with EPN occurrence maps to produce correlation maps, allowing spatial inference of potential relationships between EPNs and specific soil characteristics. Model performance was evaluated using cross-validation procedures, and prediction errors were assessed through RMSE values.

Statistical analyses were performed using Minitab® software (version 22), where correlation tests were first performed to assess relationships between variables. Subsequently, simple linear regression models were fitted to explore relationships between EPN presence and soil chemical attributes, and the resulting equations were derived from these relationships. Given that EPN occurrence was treated as a binary variable, the regression approach should be interpreted as exploratory rather than predictive. Only statistically significant relationships (*p* < 0.05) were considered.

To further investigate the multivariate relationships among soil chemical attributes, a Principal Component Analysis (PCA) was performed using standardized (z-score) variables, including Al, Ca, Mg, K, Mn, S, B, Fe, Cu, Zn, and base saturation (V%). Standardization was applied because the variables were measured in different units and scales, ensuring that each variable contributed equally to the analysis. PCA was based on the correlation matrix, and principal components were extracted through eigenvalue decomposition. The first two principal components were retained for graphical interpretation because they explained most of the total variance in the dataset, providing an effective representation of the main gradients of soil chemical variability. PCA was used as an exploratory multivariate technique to identify associations among soil chemical attributes and to evaluate whether EPN occurrence was related to broader patterns of soil chemistry rather than to isolated variables.

Model performance was evaluated using cross-validation. The predictive accuracy of interpolated surfaces was assessed through Root Mean Square Error (RMSE), calculated by comparing observed and predicted values.

## Results

Table 3 presents the regression models fitted in Minitab®, describing the relationships between entomopathogenic nematode (EPN) presence and soil chemical attributes. Significant associations (*p* < 0.05) were observed for potassium (K), sulfur (S), boron (B), iron (Fe), copper (Cu), and zinc (Zn).

**Table 3:** Simple linear regression results between entomopathogenic nematode (EPN) presence and soil chemical attributes

Nutrient	Correlation/*p*-value	Model	*R*^2^ (%)	RMSE (%)
Ca	0.10/0.45	60.3 + 3.15 Ca	0.9	21.78
Mg	0.29/0.07	−54.1 + 85.1 Mg	3.6	21.03
K	0.68/0.00	−12.8 + 35.2 K	44.4	16.17
Mn	0.23/0.08	57.1 + 3.57 Mn	3.4	21.32
S	0.60/0.00	−49.7 + 4.88 S	33.3	17.72
B	0.78/0.00	−115 + 188 B	59.9	13.76
Fe	0.67/0.00	−1.1 + 2.58 Fe	42.2	16.48
Cu	0.40/0.02	34.6 + 3.30 Cu	13.6	20.33
Zn	0.50/0.00	48.0 + 0.86 Zn	19.6	19.45

Among these variables, boron showed the strongest relationship with EPN occurrence (*r* = 0.78, *p* = 0.00; *R*^2^ = 59.9%; RMSE = 13.76), followed by potassium (*r* = 0.68, *p* = 0.00; *R*^2^ = 44.4%; RMSE = 16.17) and iron (*r* = 0.67, *p* = 0.00; *R*^2^ = 42.2%; RMSE = 16.48). Sulfur also showed a moderate association (*r* = 0.60, *p* = 0.00; *R*^2^ = 33.3%; RMSE = 17.72), while zinc (*r* = 0.50, *p* = 0.00; *R*^2^ = 19.6%; RMSE = 19.45) and copper (*r* = 0.40, *p* = 0.02; *R*^2^ = 13.6%; RMSE = 20.33) presented weaker, although significant, relationships.

In contrast, calcium (*r* = 0.10, *p* = 0.45; *R*^2^ = 0.9%; RMSE = 21.78), magnesium (*r* = 0.29, *p* = 0.07; *R*^2^ = 3.6%; RMSE = 21.03), and manganese (*r* = 0.23, *p* = 0.08; *R*^2^ = 3.4%; RMSE = 21.32) showed no significant association with EPN occurrence and low explanatory power. These statistical results were partially supported by spatial analysis patterns, which revealed consistent patterns for certain nutrients.

Spatial analysis partially supported the regression results. Higher EPN densities were consistently observed in areas with elevated potassium and iron concentrations across both farms. In contrast, copper showed an opposite pattern, with lower EPN occurrence in areas of higher Cu content (Figs. 3, 4, 5, 6, 7, 8, 9, 10, 11, 12, 13, and 14).

**Fig. 3:**
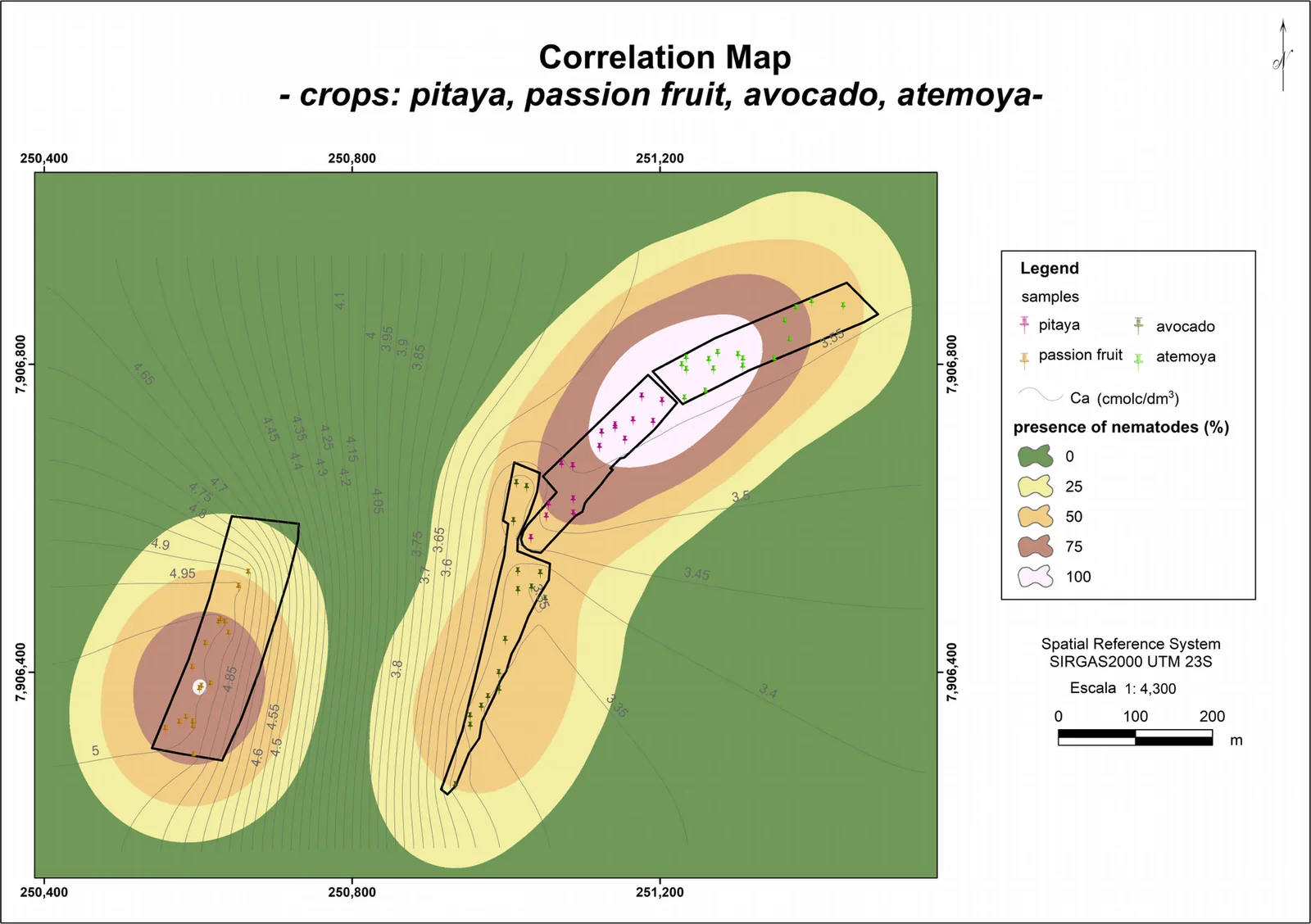
Correlation map—crops: pitaya, passion fruit, avocado, atemoya—Ca (cmolc/dm^3^)

**Fig. 4:**
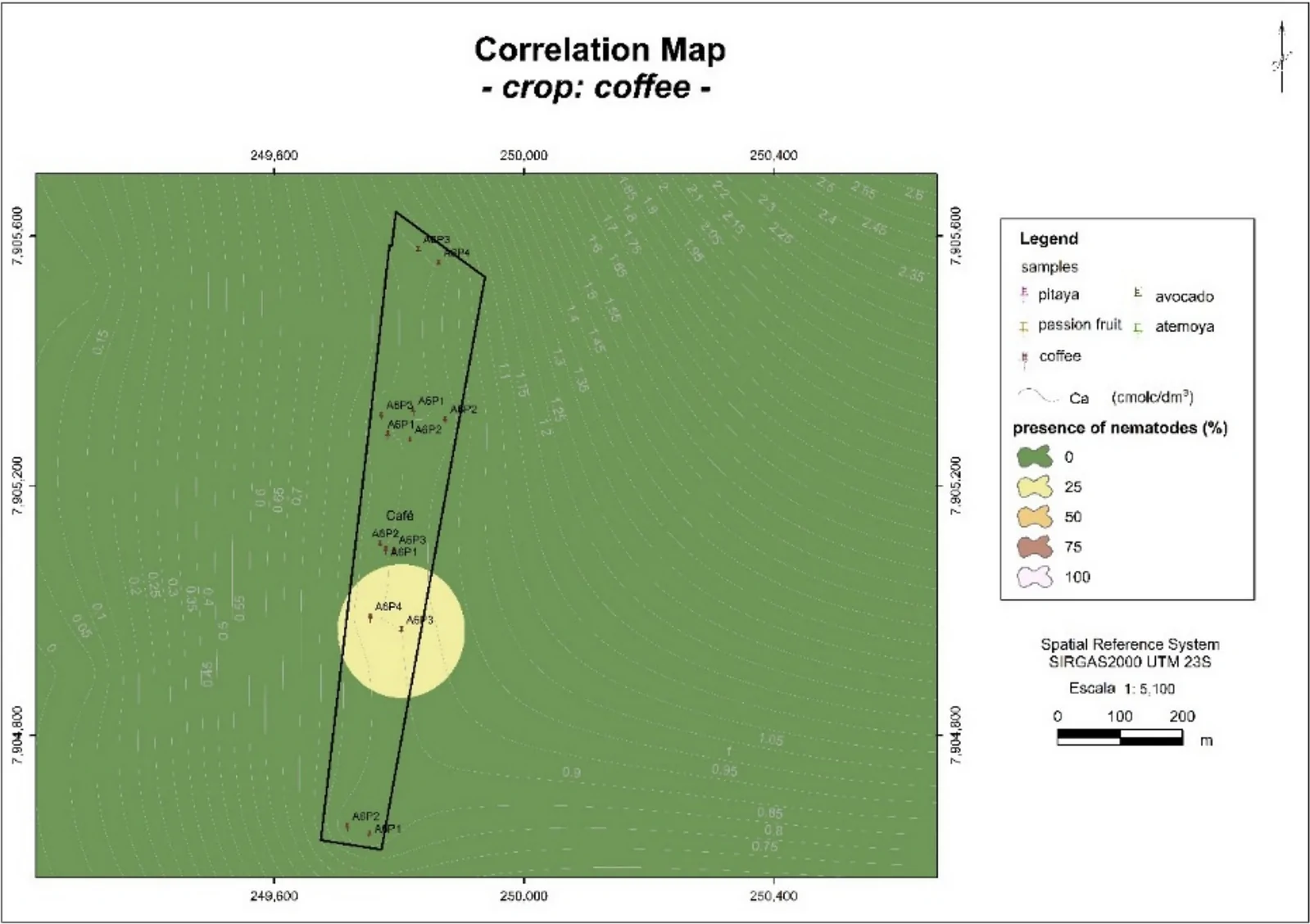
Correlation map—crop: coffee—Ca (cmolc/dm^3^)

**Fig. 5:**
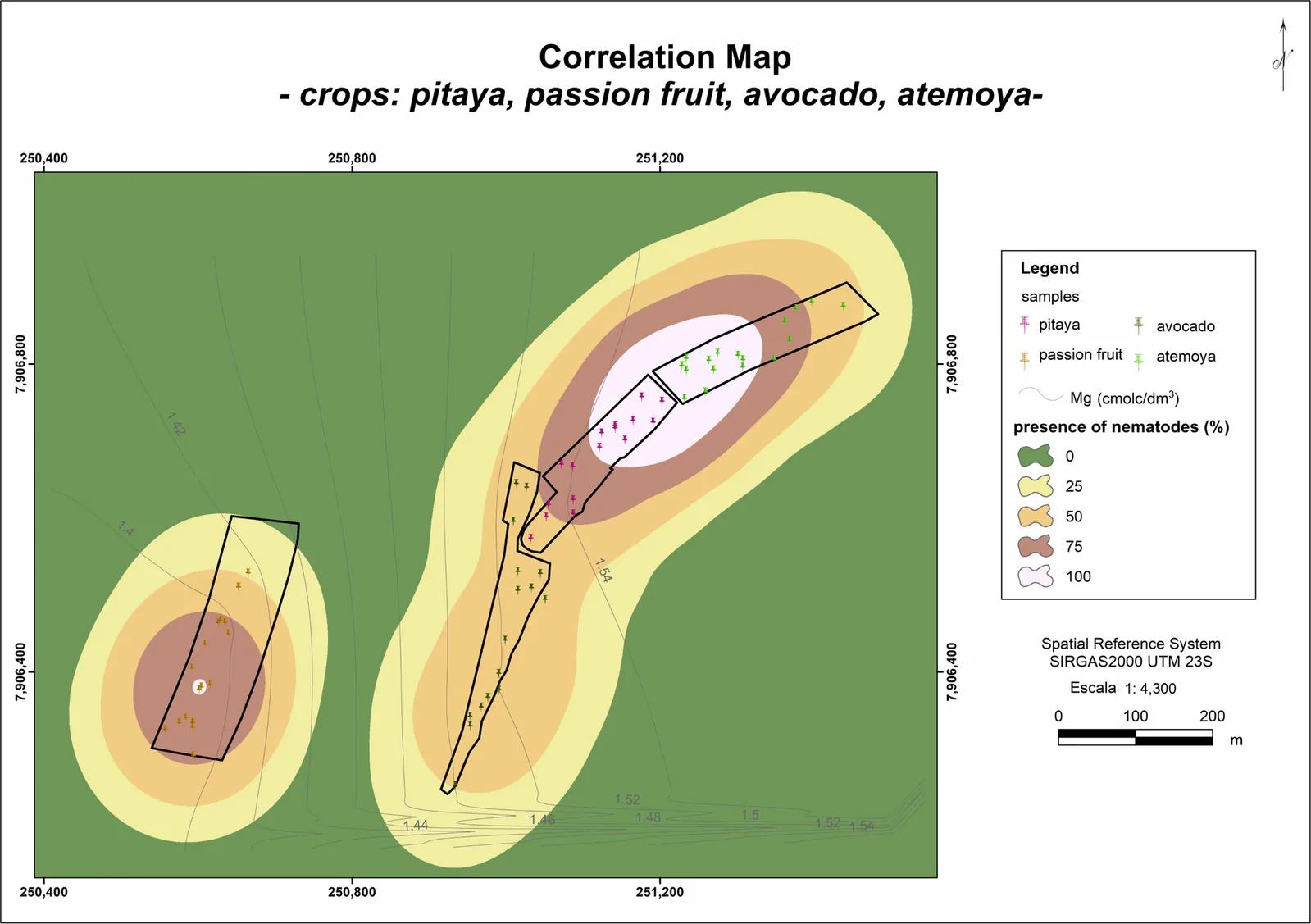
Correlation map—crops: pitaya, passion fruit, avocado, atemoya—Mg (cmolc/dm^3^)

**Fig. 6:**
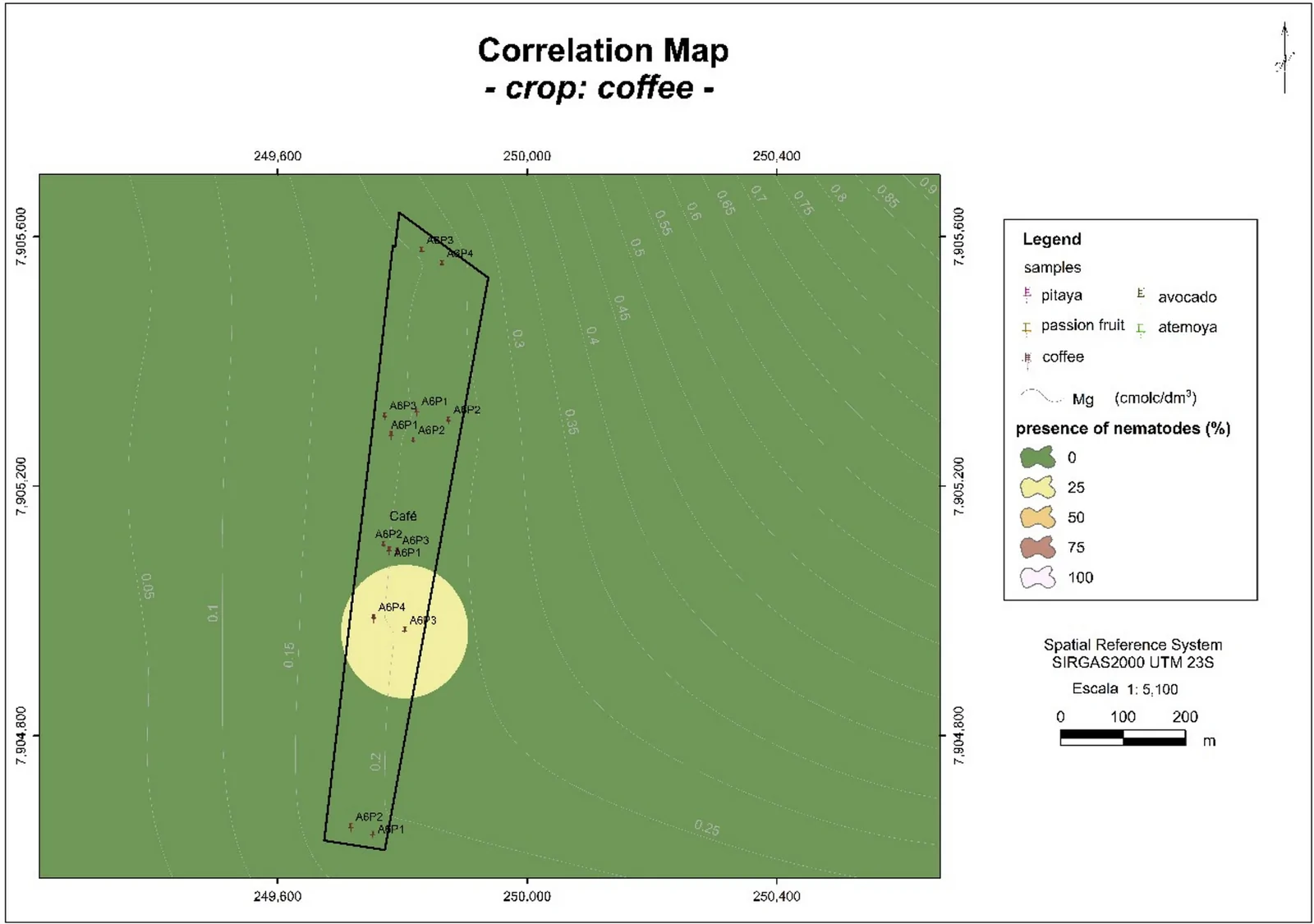
Correlation map—crop: coffee—Mg (cmolc/dm^3^)

**Fig. 7:**
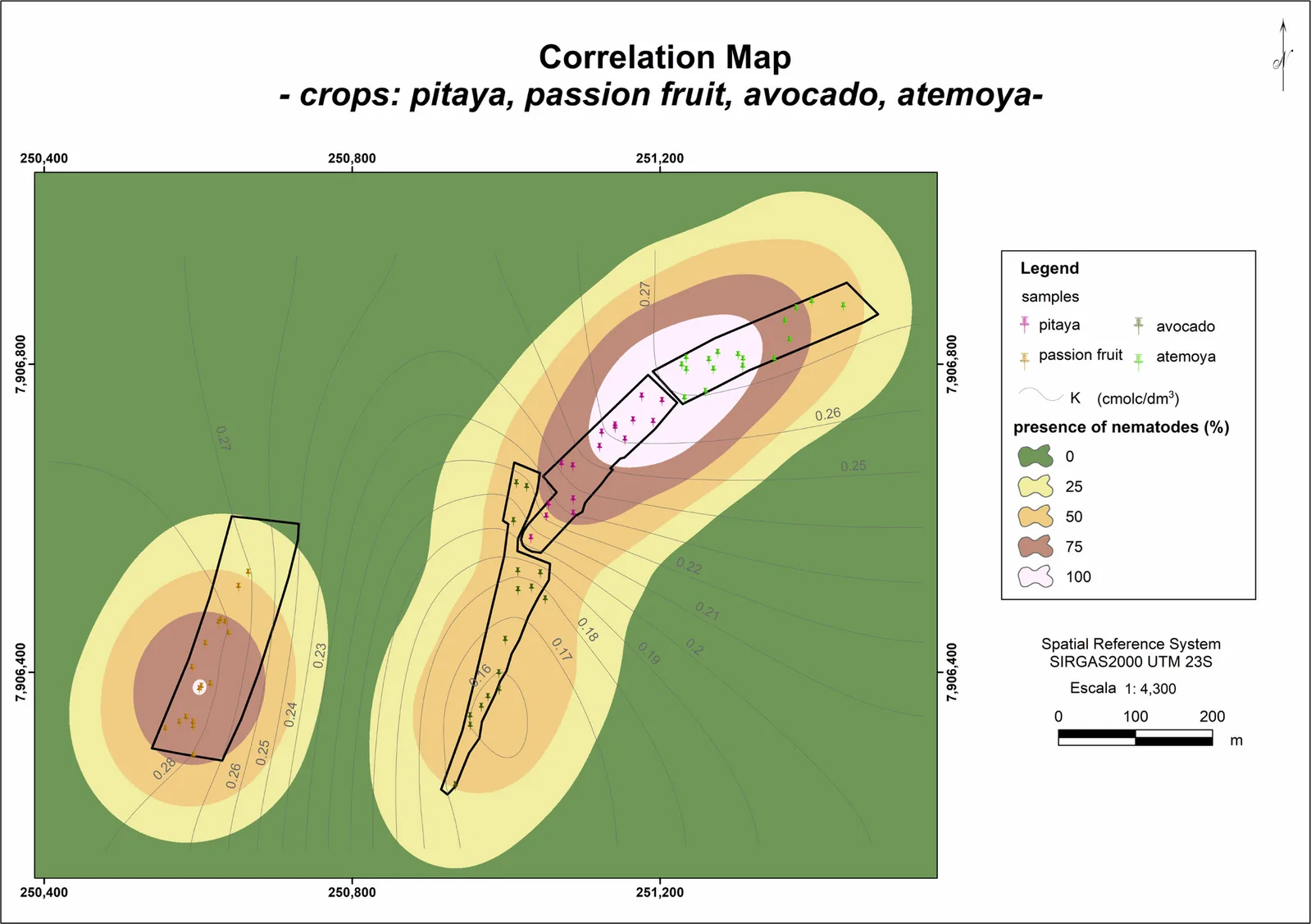
Correlation map— crops: pitaya, passion fruit, avocado, atemoya—K (cmolc/dm^3^)

**Fig. 8:**
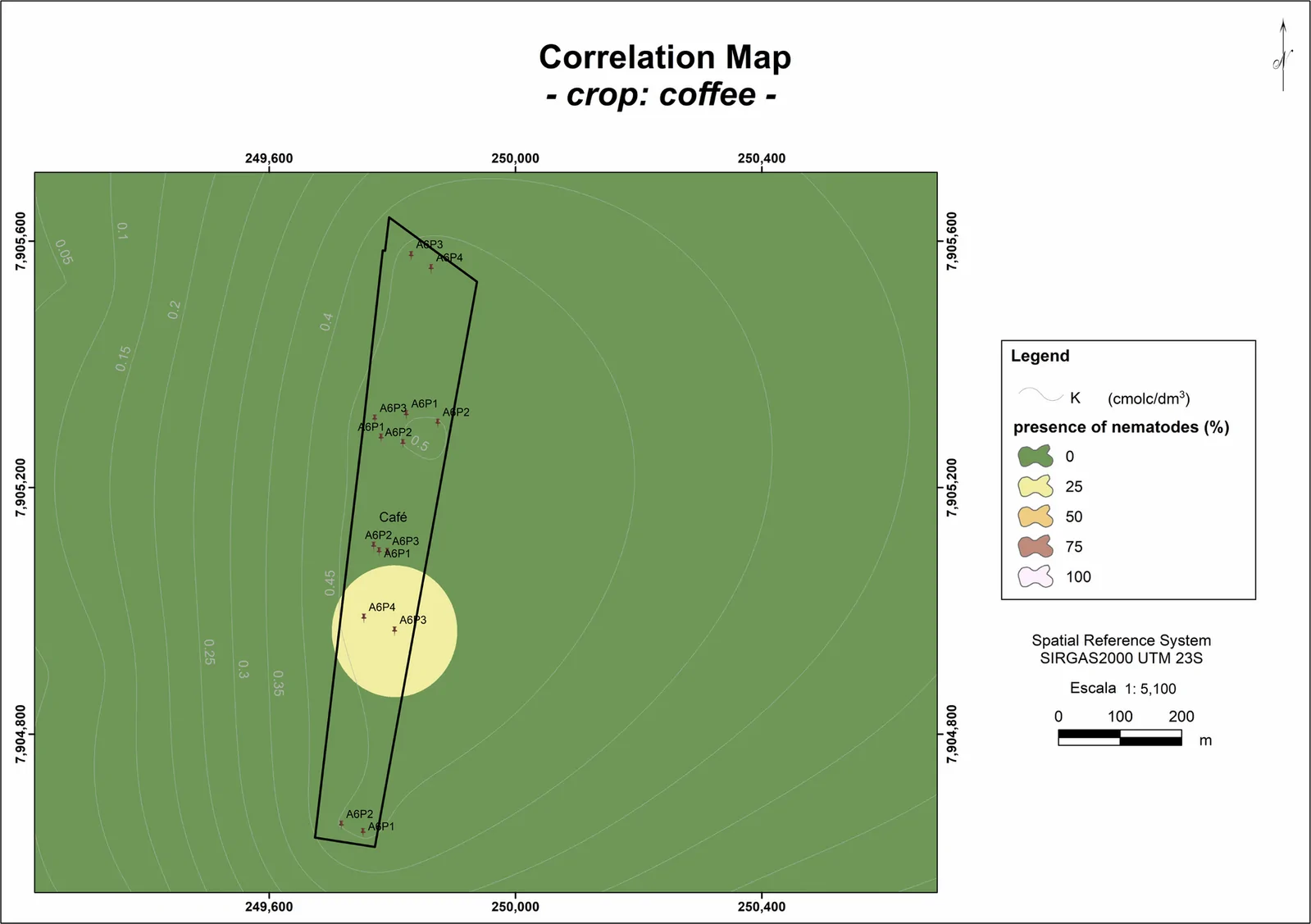
Correlation map—crop: coffee—K (cmolc/dm^3^)

**Fig. 9:**
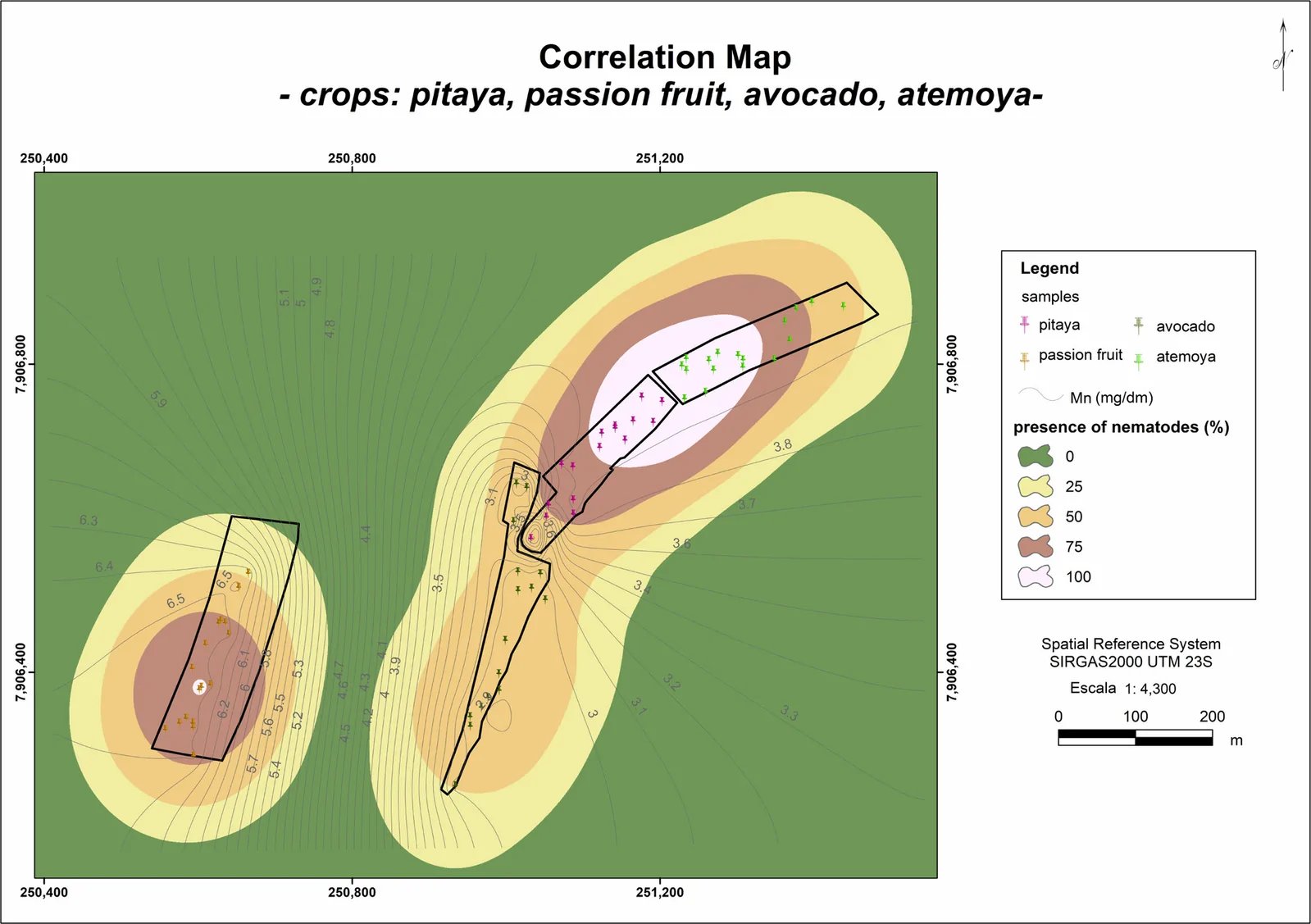
Correlation map—crops: pitaya, passion fruit, avocado, atemoya—Mn (mg/dm^3^)

**Fig. 10:**
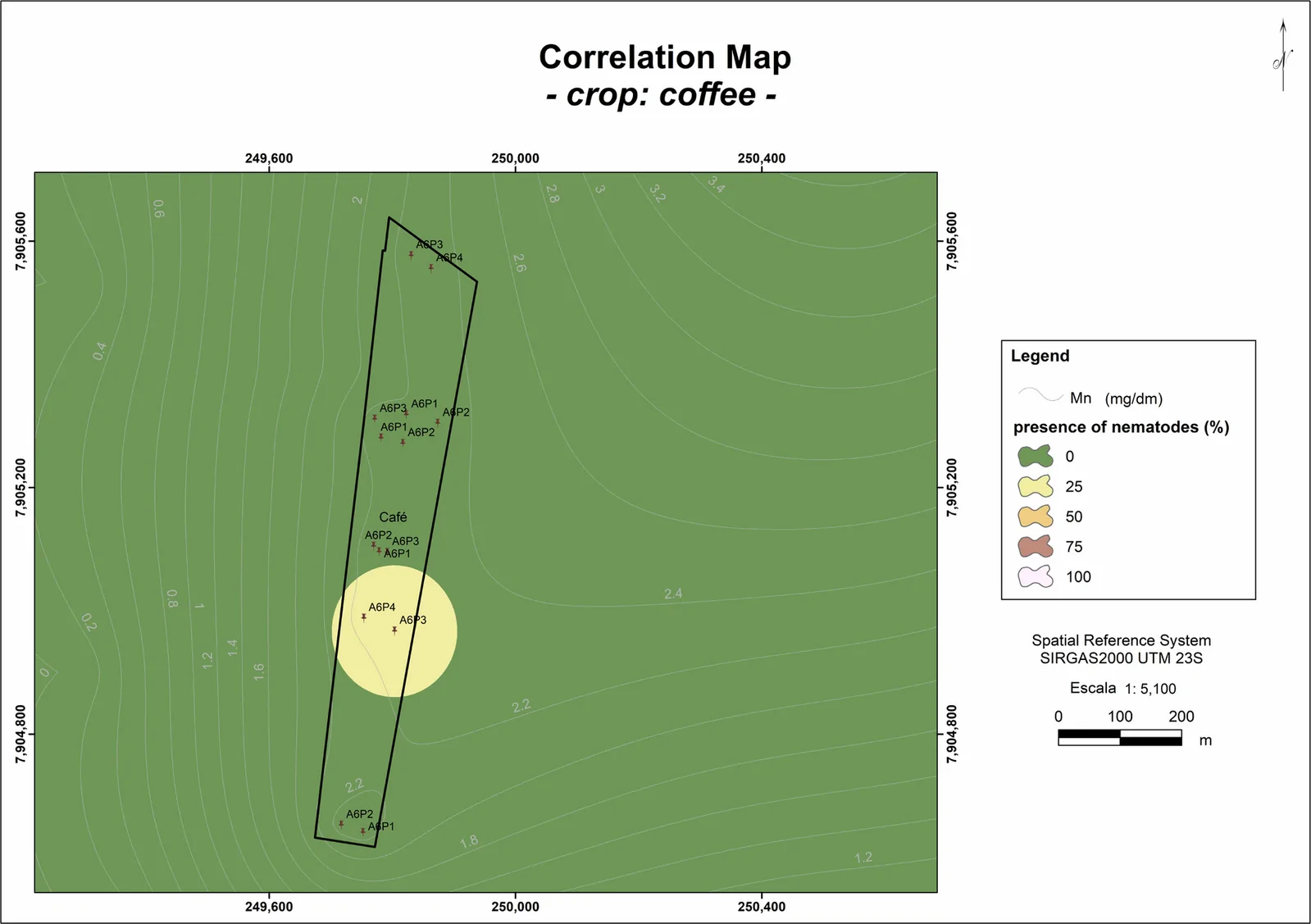
Correlation map—crop: coffee—Mn (mg/dm^3^)

**Fig. 11:**
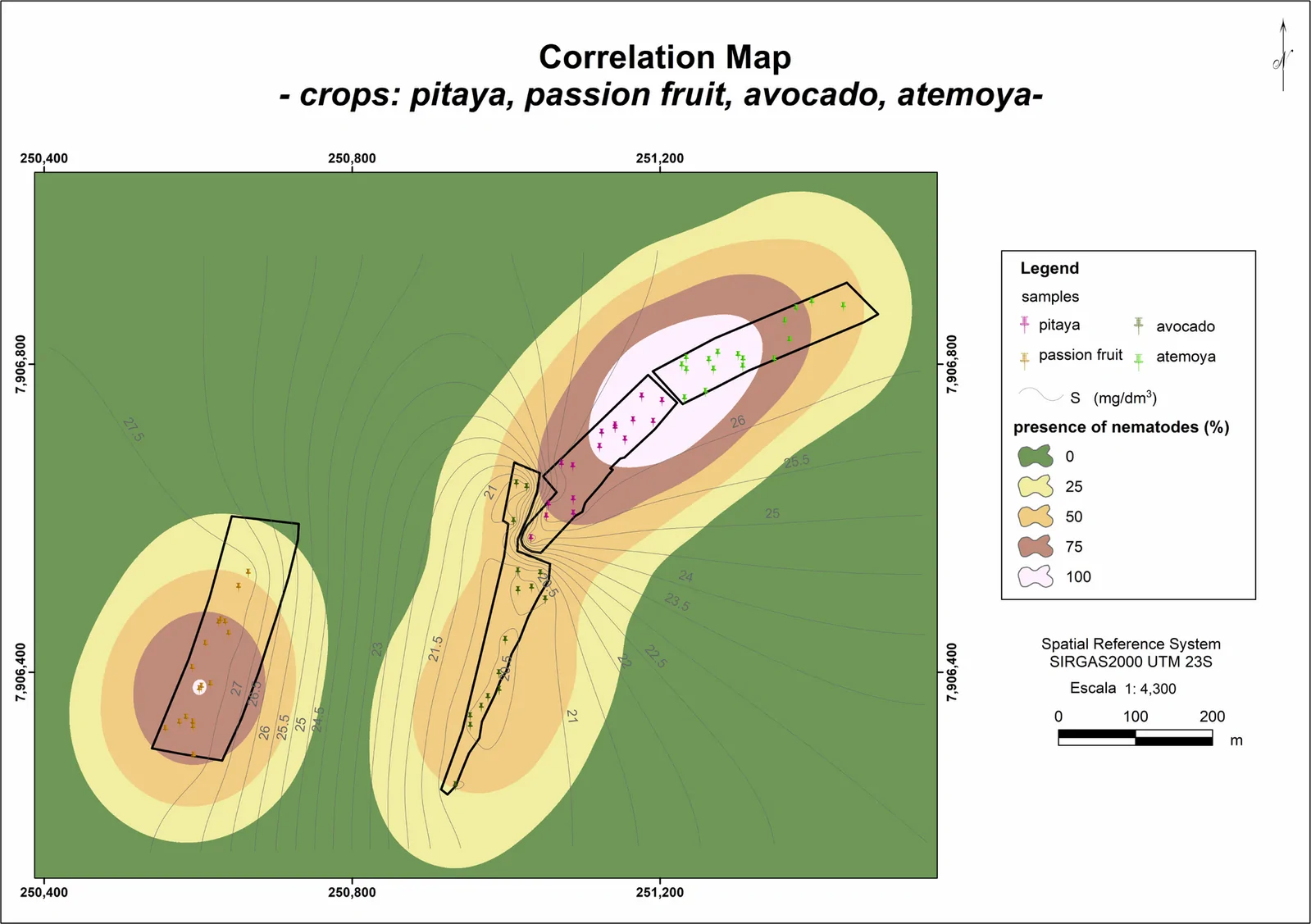
Correlation map—crops: pitaya, passion fruit, avocado, atemoya—S (mg/dm^3^)

**Fig. 12:**
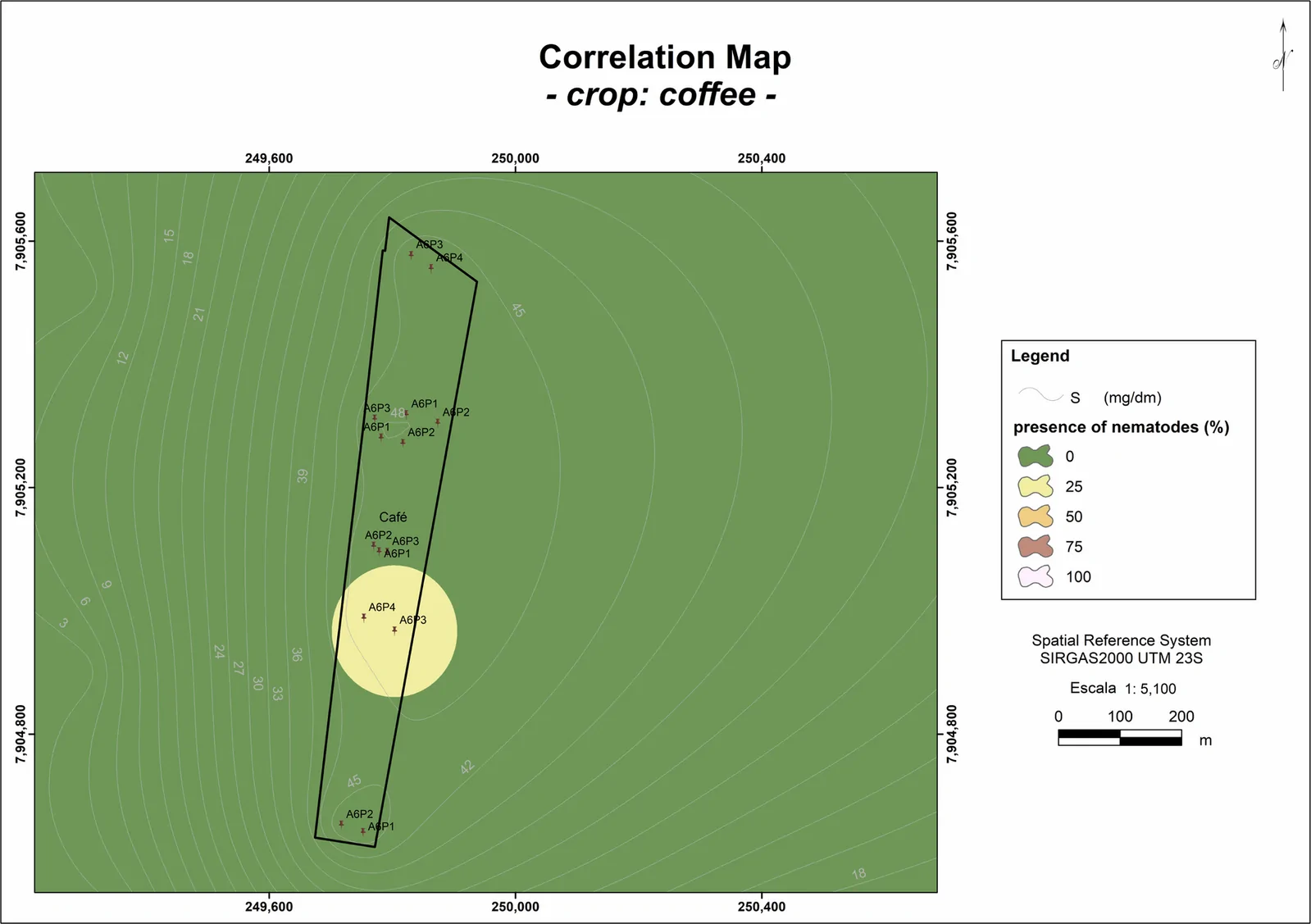
Correlation map—crop: coffee—S (mg/dm^3^)

**Fig. 13:**
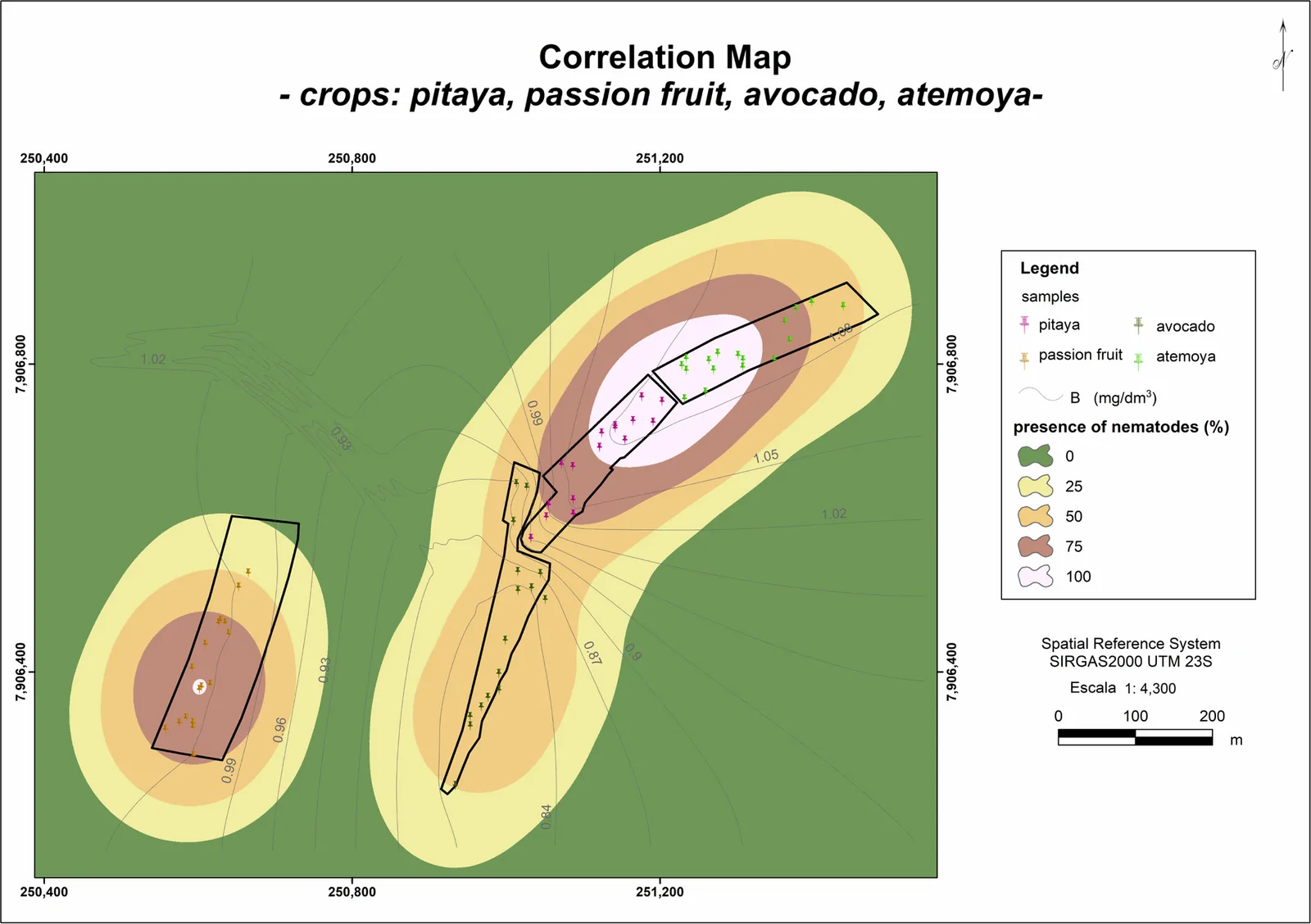
Correlation map—crops: pitaya, passion fruit, avocado, atemoya—B (mg/dm^3^)

**Fig. 14:**
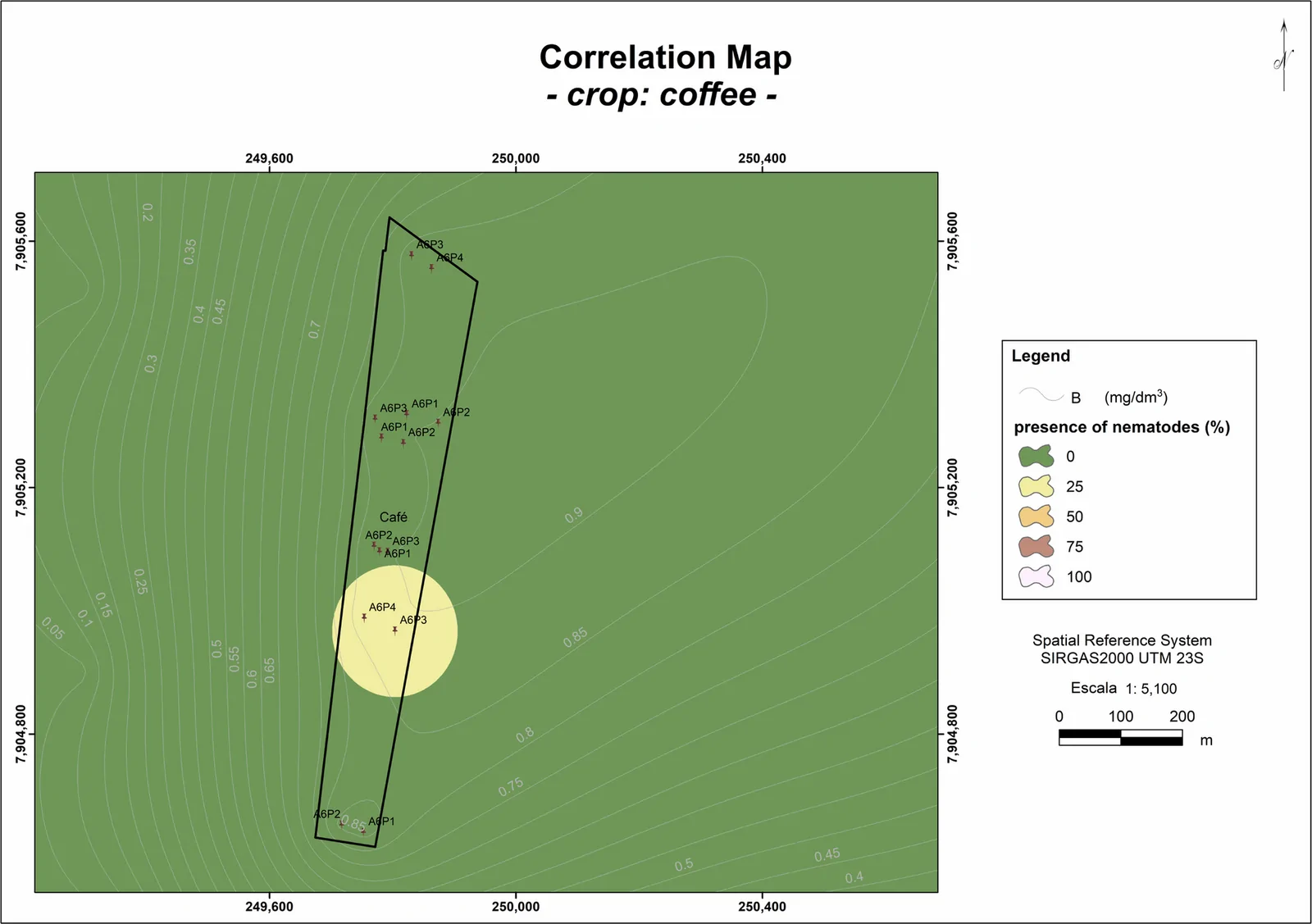
Correlation map—crop: coffee—B (mg/dm^3^)

For zinc, spatial distribution varied between farms, with EPN presence associated with higher concentrations at Pastão and intermediate levels at Londrina. Boron did not show a consistent spatial pattern despite its strong statistical association. Likewise, calcium, magnesium, sulfur, and manganese exhibited heterogeneous distributions and no clear spatial relationship with EPN occurrence.

**Fig. 15:**
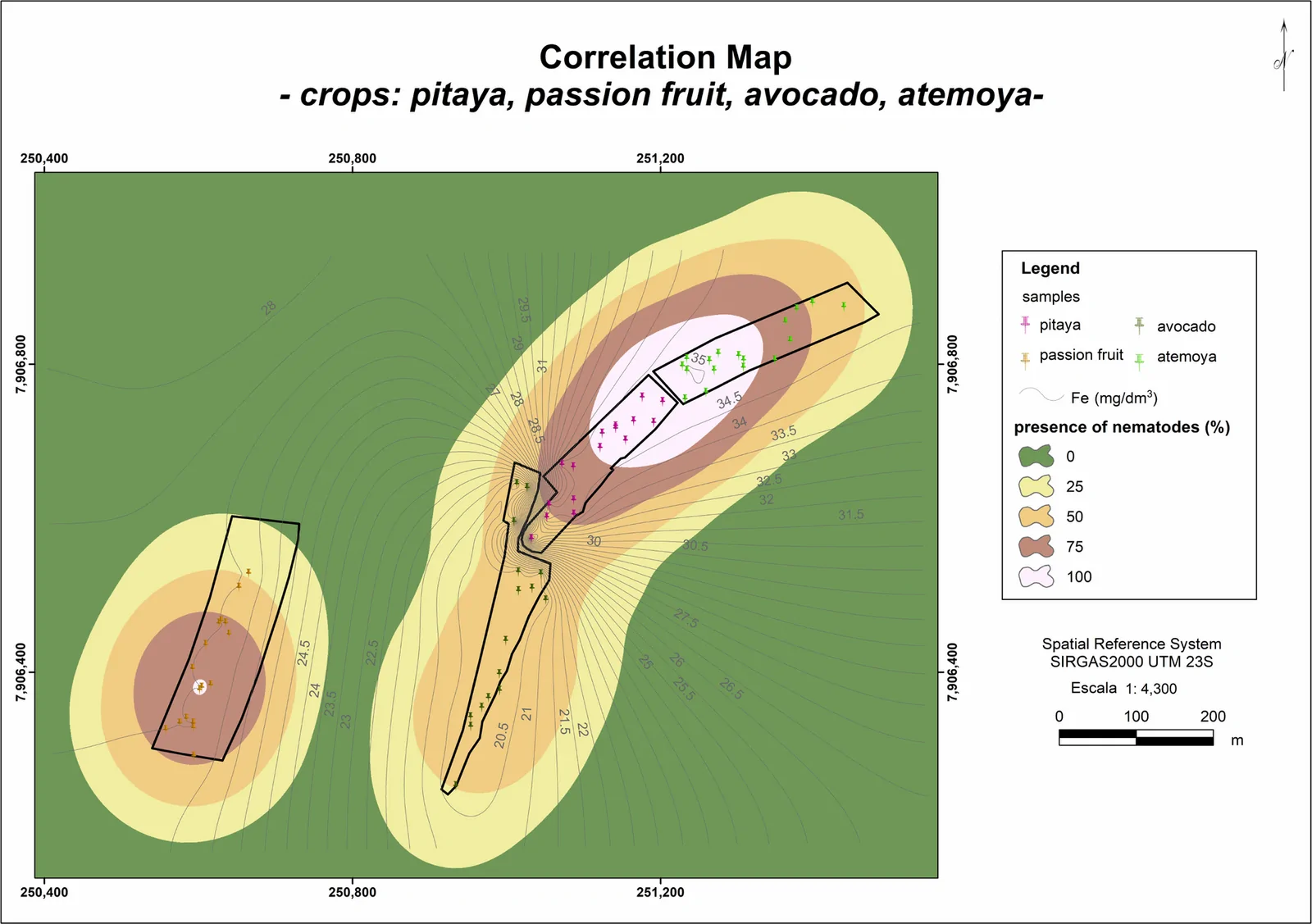
Correlation map—crops: pitaya, passion fruit, avocado, atemoya—Fe (mg/dm^3^)

**Fig. 16:**
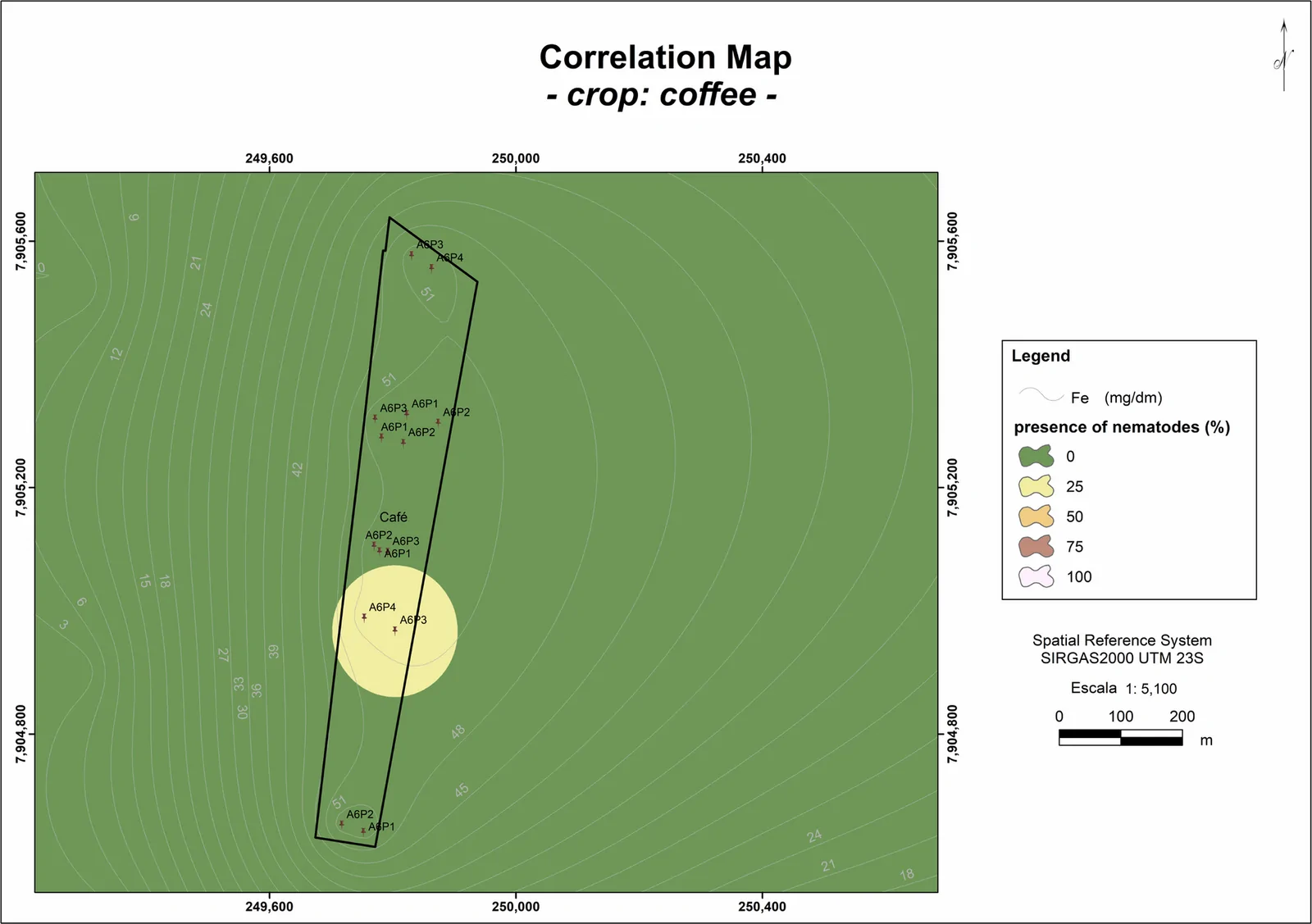
Correlation map—crop: coffee—Fe (mg/dm^3^)

**Fig. 17:**
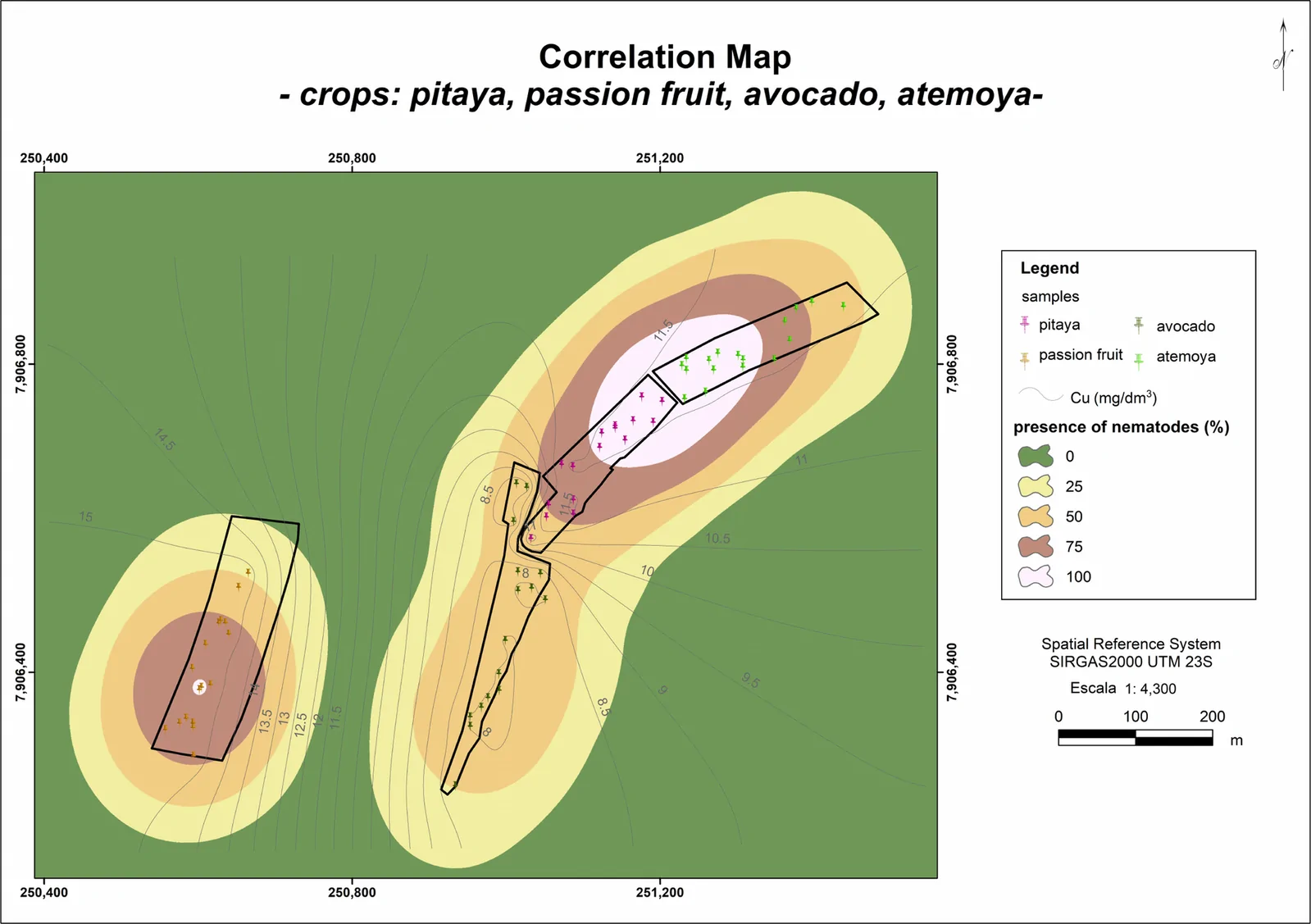
Correlation map—crops: pitaya, passion fruit, avocado, atemoya—Cu (mg/dm^3^)

**Fig. 18:**
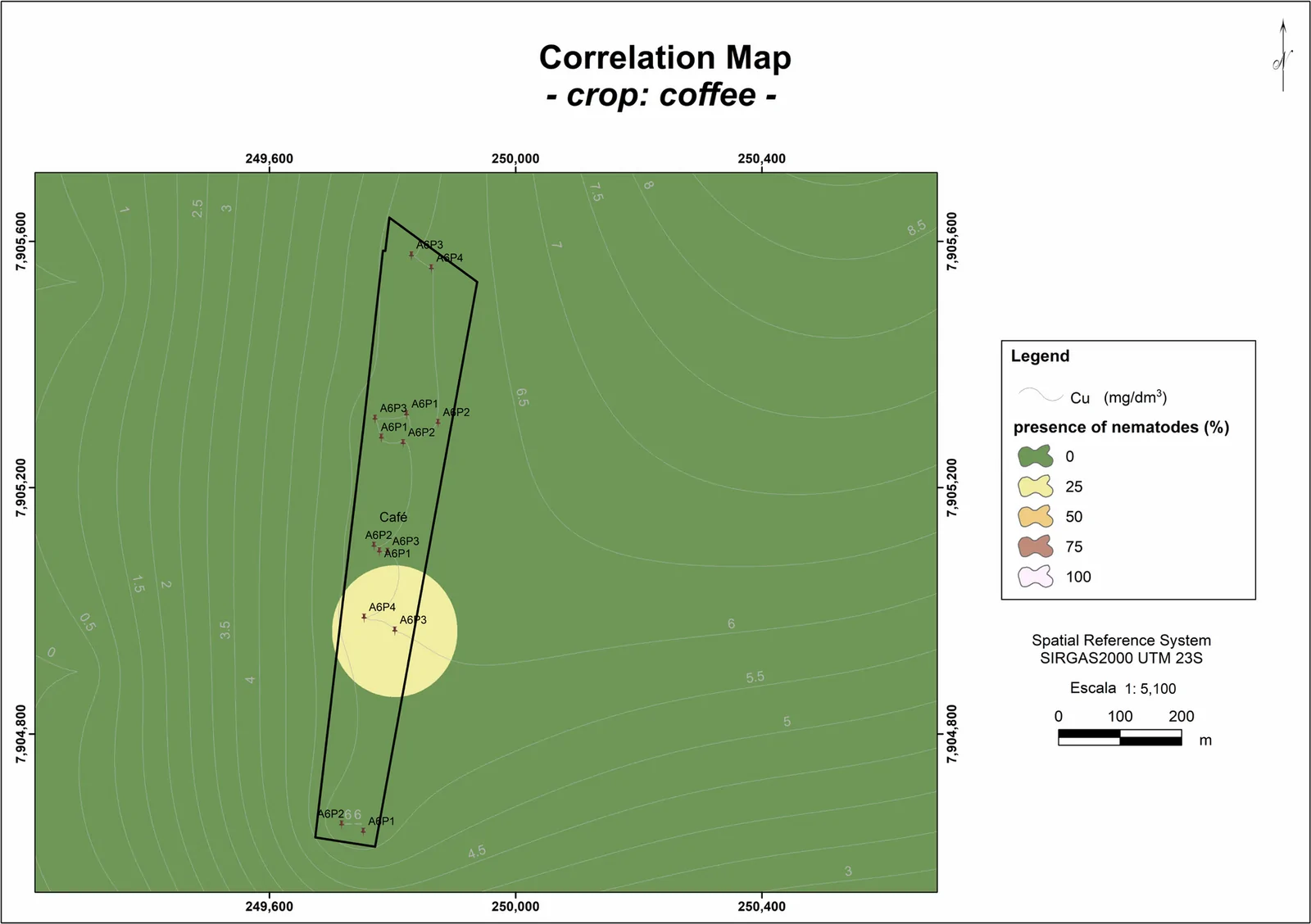
Correlation map—crop: coffee—Cu (mg/dm^3^)

**Fig. 19:**
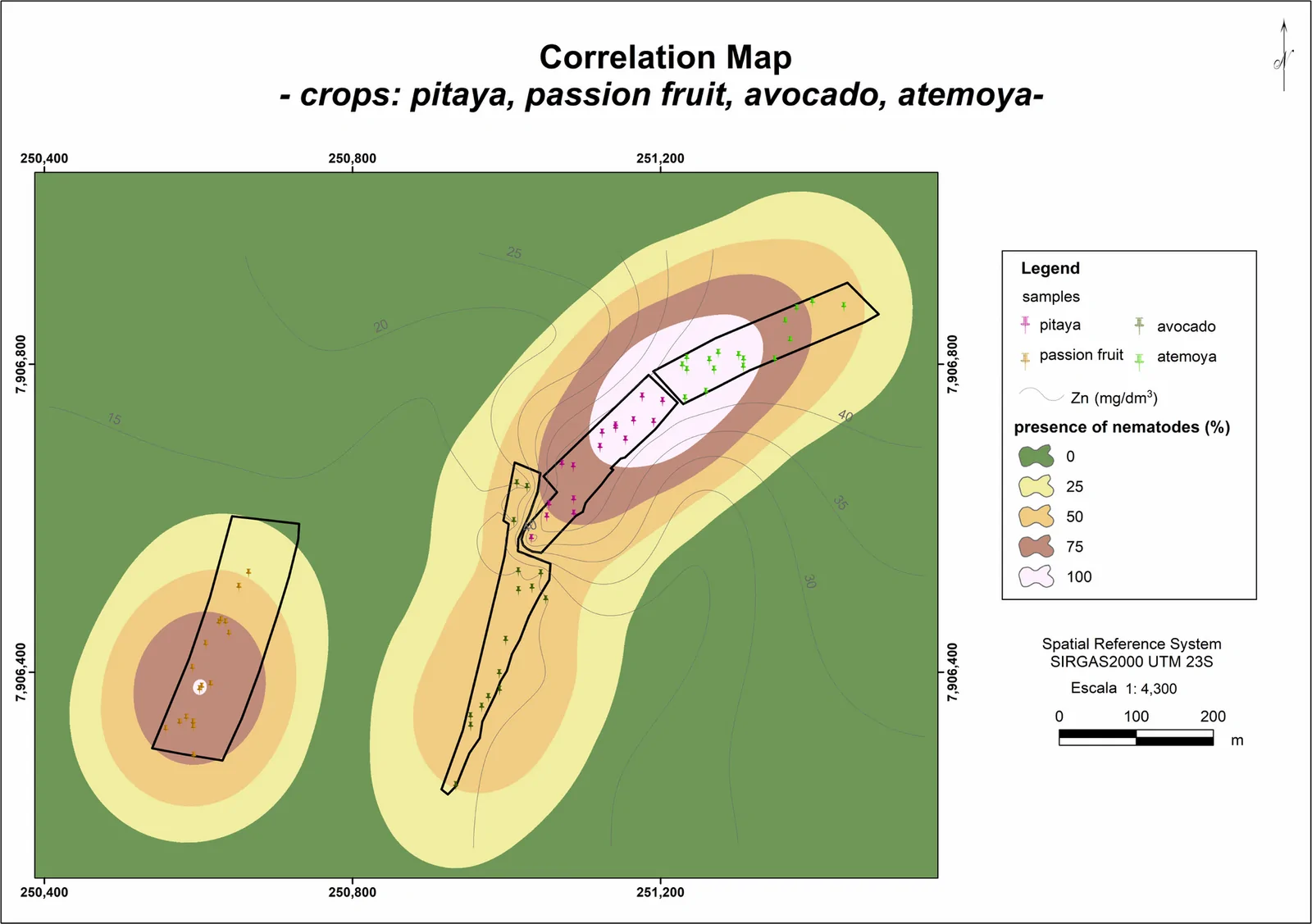
Correlation map—crops: pitaya, passion fruit, avocado, atemoya—Zn (mg/dm^3^)

**Fig. 20:**
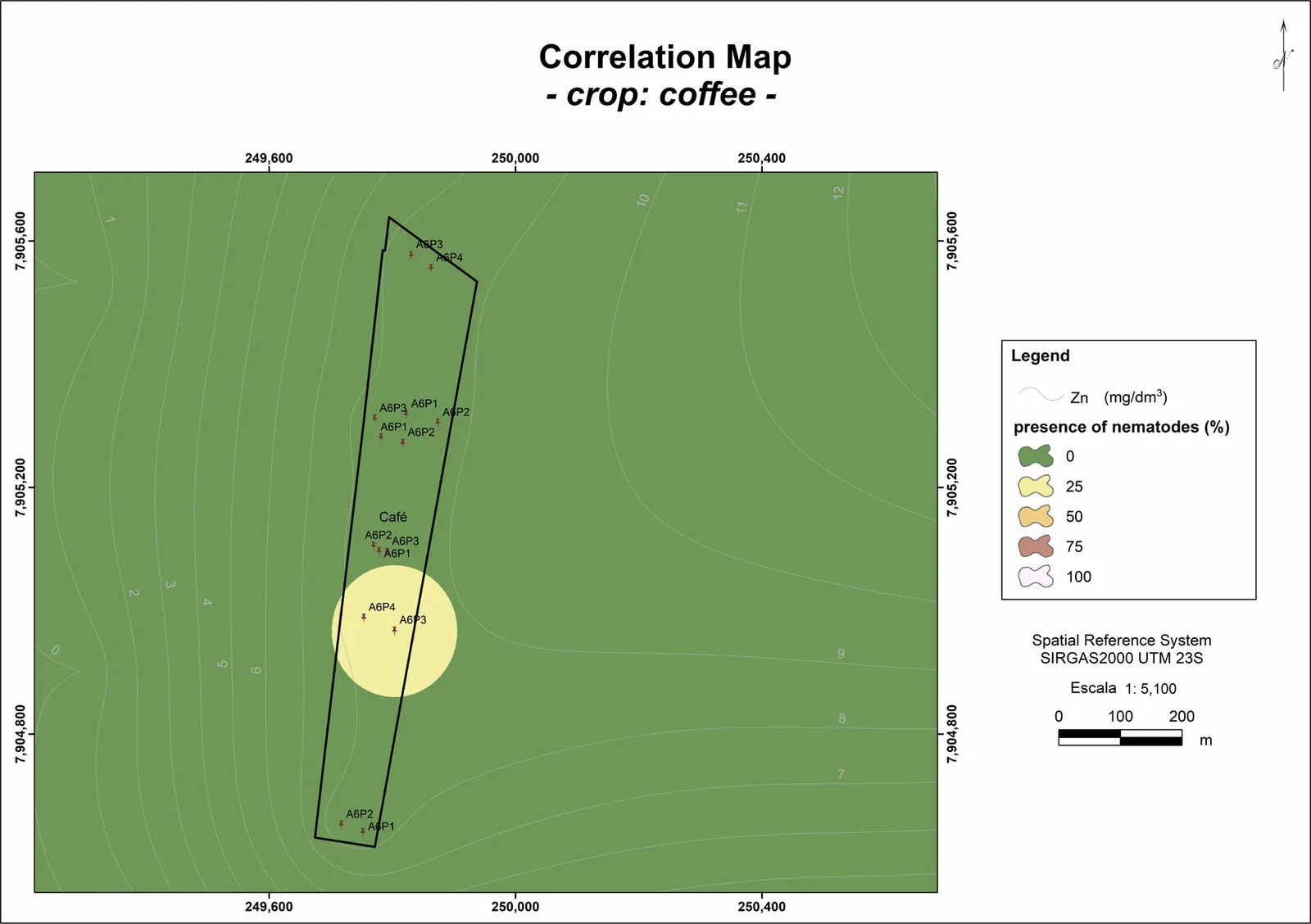
Correlation map—crop: coffee—Zn (mg/dm^3^)

At both farms, EPNs were more frequently detected in areas with potassium concentrations above 0.23 cmolc/dm^3^ and in zones with elevated iron levels (Figs. 7, 8, 15, and 16). Conversely, EPN occurrence tended to decrease in areas with higher copper concentrations, particularly at Pastão farm (Figs. 17 and 18). Zinc displayed contrasting patterns between farms, preventing the identification of a consistent threshold associated with EPN presence (Figs. 19 and 20).

For calcium and magnesium, EPN occurrence was observed across a broad range of concentrations without a consistent trend. Similarly, sulfur and boron showed no apparent spatial relationship with nematode distribution, while manganese exhibited only a weak tendency toward greater EPN occurrence in areas with higher nutrient concentrations (Figs. 3, 4, 5, 6, 7, 8, 9, 10, 11, 12, 13, and 14). Overall, these results indicate that potassium and iron were the nutrients most consistently associated with EPN occurrence, whereas the remaining soil attributes showed variable or inconclusive spatial patterns.

**Fig. 21:**
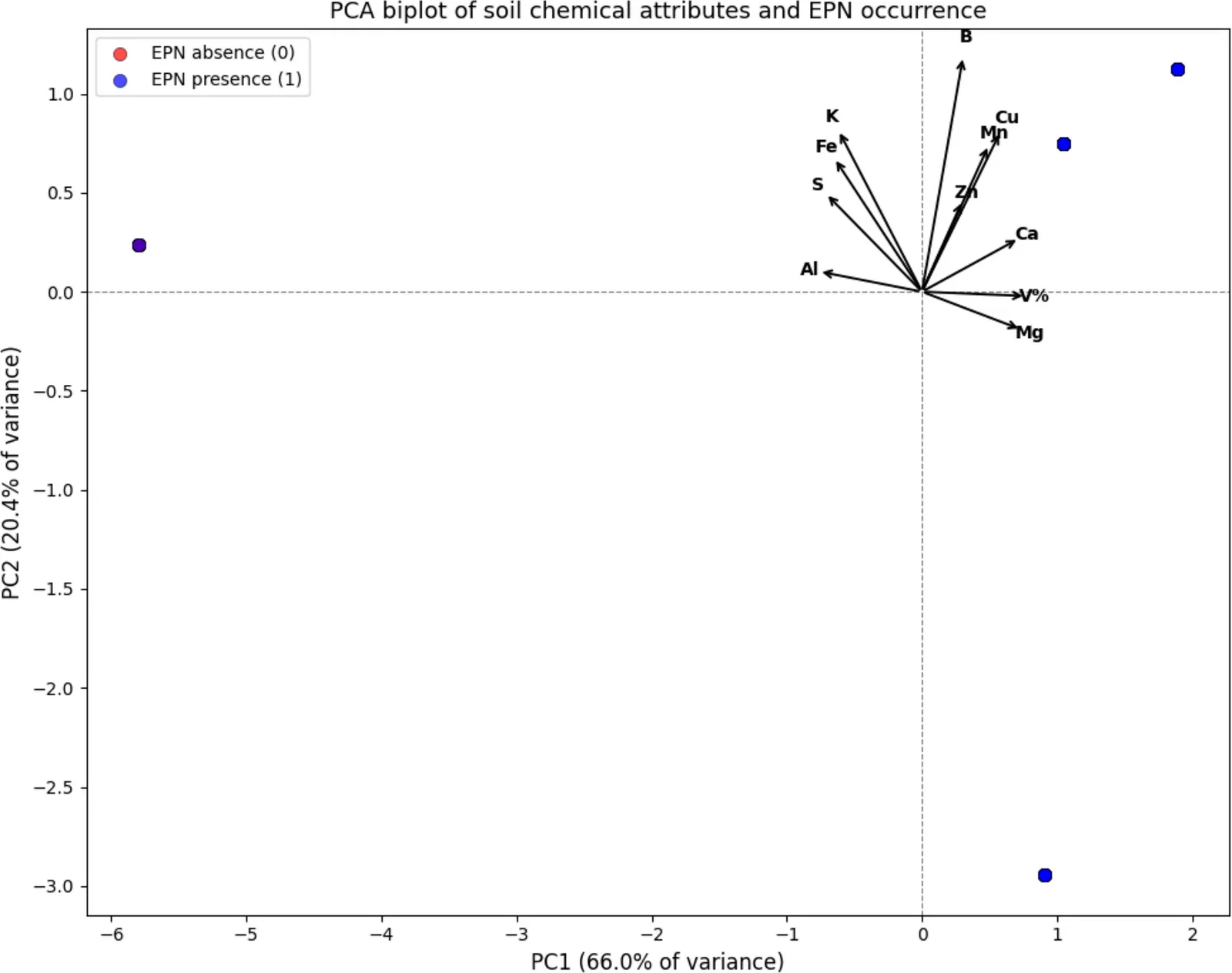
Principal Component Analysis (PCA) biplot of the soil chemical attributes (Al, Ca, Mg, K, Mn, S, B, Fe, Cu, Zn, and base saturation, V%) and their relationship with entomopathogenic nematode (EPN) occurrence. Blue circles represent samples with EPN presence (1), whereas red circles represent samples with EPN absence (0). Arrows indicate the direction and magnitude of the contribution (loadings) of each soil chemical attribute to the first two principal components (PC1 and PC2), which together explained 86.34% of the total variance

Principal Component Analysis (PCA) revealed that the first two principal components explained 86.4% of the total variance, with PC1 accounting for 66.0% and PC2 for 20.4%. PC1 mainly represented a gradient separating soils with higher concentrations of calcium, magnesium, copper, manganese, zinc, boron, and higher base saturation (V%) from soils characterized by higher aluminum, sulfur, iron, and potassium contents (Fig. 21). The PCA confirmed that soil chemical attributes were strongly interrelated, indicating that EPN occurrence is associated with multivariate soil conditions rather than isolated chemical properties.

## Discussion

The positive association observed between potassium and iron and EPN occurrence suggests that soil fertility may indirectly regulate nematode populations through plant–microbe interactions in the rhizosphere. Potassium is known to enhance root development and exudation patterns, stimulating microbial activity and potentially creating environments that favor EPN survival and host-seeking behavior (Li et al., 2024). Similarly, iron plays a central role in microbial competition and metabolism, particularly through siderophore-mediated processes that influence microbial community structure (Gu et al., 2020; Hu et al., 2024). As EPNs depend on symbiotic bacteria such as Xenorhabdus and Photorhabdus for infectivity, changes in microbial dynamics may directly affect their performance and persistence in soil.

Copper, in contrast, exhibited a negative association with EPN occurrence. Elevated Cu concentrations are known to alter soil microbial communities, reducing diversity and functional activity, which are essential for maintaining ecological processes in the soil (Campillo-Cora et al., 2025; Navarrete et al., 2017). Given the close relationship between EPNs and their symbiotic bacteria, copper-induced shifts in microbial structure may indirectly limit nematode survival and infectivity. This pattern reinforces the role of trace metals as important regulators of soil biological processes and highlights the potential negative impacts of excessive copper accumulation in agricultural systems.

Zinc also displayed variable responses across farms, preventing the identification of a clear ecological threshold associated with EPN occurrence. Although zinc is an essential micronutrient and has been reported to influence the functional structure of soil microbial communities (Koppenhöfer & Fuzy, 2006), its effects on entomopathogenic nematodes remain poorly understood. The inconsistent distribution patterns observed in this study suggest that the influence of Zn on EPNs is likely indirect and context dependent, possibly mediated by changes in microbial activity and nutrient cycling processes. In contrast, calcium, magnesium, sulfur, manganese, and boron did not exhibit consistent spatial relationships with EPN occurrence across the study sites. These nutrients play important roles in soil fertility, aggregation, nutrient cycling, and microbial activity, but their effects on EPN ecology are likely indirect and strongly influenced by local environmental conditions. For example, calcium and magnesium contribute to soil structure and cation exchange processes that may affect nematode mobility, whereas sulfur can alter soil pH through oxidative processes, indirectly influencing microbial community composition (Schroder & Fouss, 1992). Similarly, manganese availability is highly dependent on soil pH and redox conditions, while elevated boron concentrations may affect microbial respiration and diversity. The absence of consistent spatial patterns for these elements suggests that EPN distribution is not governed by individual nutrients alone but rather by the interaction of multiple soil properties.

The variability observed among farms further emphasizes the importance of site-specific factors such as crop type, management practices, rhizosphere dynamics, and microclimatic conditions in shaping EPN populations. Together, these findings indicate that EPN distribution is regulated by a complex network of interactions involving soil chemistry, microbial communities, and plant–soil processes. Consequently, agronomic thresholds established for crop nutrition may not necessarily correspond to optimal conditions for beneficial soil organisms. This highlights the importance of adopting integrative approaches when evaluating the ecological drivers of biological control agents in agricultural soils, particularly under tropical conditions where environmental heterogeneity is high.

It is important to emphasize that the relationships identified in this study are based on spatial correlations and therefore do not establish causal mechanisms. In addition, the absence of aluminum (Al^3^⁺) measurements for one of the study areas limited our ability to evaluate the potential influence of soil acidity–related toxicity on EPN persistence. Previous studies have suggested that acidic conditions may reduce EPN survival and infectivity through increased aluminum solubility (Matuska-Łyżwa et al., 2024). Future studies combining controlled experiments with multivariate approaches will be necessary to disentangle the relative contribution of soil chemical, biological, and management factors and improve our understanding of the mechanisms governing EPN distribution in agricultural soils.

Overall, the results highlight that EPN distribution is governed by a complex interplay of soil chemical and biological factors rather than by isolated nutrient effects. The associations observed here likely reflect indirect mechanisms mediated by plant physiology and microbial interactions in the rhizosphere. While causal relationships cannot be established from this study, the integration of spatial analysis with soil chemical data provides valuable insights into the ecological drivers of EPN occurrence. Principal Component Analysis (PCA) complemented the regression analyses by demonstrating that soil chemical attributes were organized along well-defined multivariate gradients rather than acting independently. This finding reinforces the interpretation that EPN occurrence is associated with the combined influence of multiple soil properties, and that the significant relationships observed for potassium and iron should be interpreted within the broader context of soil chemical interactions. Future studies integrating multivariate approaches with controlled experimental designs will be essential to disentangle these complex interactions and improve the predictability of biological control performance in agroecosystems.

## Limitations and future perspectives

This study was based on an observational field survey conducted under commercial farming conditions. Therefore, the spatial associations identified between entomopathogenic nematodes (EPNs) and soil chemical attributes should not be interpreted as evidence of causal relationships. Multiple environmental factors, including crop management, soil moisture, texture, pH, microbial community composition, and climatic conditions, may simultaneously influence EPN occurrence and were not fully controlled in the present study.

Another limitation is that the study evaluated a single sampling period in each crop system, which represents the spatial distribution of EPNs under specific environmental conditions. Seasonal variations and long-term changes in soil properties may alter these relationships over time. Furthermore, although the PCA provided a useful multivariate overview of soil chemical variability, it remains an exploratory technique and does not establish cause-and-effect relationships among variables.

Future studies should integrate controlled experiments with repeated temporal sampling, larger datasets, and additional environmental variables, including physical and biological soil attributes. The application of multivariate statistical approaches, machine learning algorithms, and spatial predictive models may further improve the understanding of the ecological drivers governing EPN distribution. Such advances may contribute to the development of site-specific biological control strategies and support precision agriculture practices aimed at optimizing the use of entomopathogenic nematodes in tropical agroecosystems.

## Conclusions

Spatial analysis revealed that EPN occurrence was positively associated with soils presenting higher potassium and iron concentrations, whereas elevated copper levels were associated with reduced EPN occurrence. Although no consistent spatial patterns were observed for magnesium, sulfur, or boron, the results indicate that EPN distribution is influenced by the interaction of multiple soil chemical properties rather than by individual nutrients acting in isolation. Principal Component Analysis (PCA) reinforced this interpretation by demonstrating that soil chemical attributes were organized along well-defined multivariate gradients, highlighting the importance of considering the integrated effects of soil chemistry on EPN occurrence.

Geospatial analysis proved to be an effective approach for identifying hotspots of EPN occurrence and exploring their relationships with soil chemical attributes. The integration of spatial analysis with multivariate statistical techniques provided a more comprehensive understanding of the environmental factors associated with EPN distribution and demonstrated the value of combining these approaches in ecological studies.

Overall, this study advances the understanding of the ecological factors associated with EPN distribution in tropical agroecosystems and provides a foundation for future research integrating spatial analysis, multivariate approaches, and controlled experimental designs. These advances may contribute to the development of more efficient, site-specific biological control strategies and support the broader adoption of EPNs within Integrated Pest Management (IPM) programs.

## Data Availability

The authors generated and analyzed original research data. All relevant data are provided within the manuscript.
